# Intratumoral heterogeneity as a potential biomarker for immunotherapy response and postoperative recurrence in NSCLC

**DOI:** 10.3389/fimmu.2026.1852284

**Published:** 2026-08-11

**Authors:** Yuhang Wang, Xiaoying Dong, Yucheng Yin, Zuju Lin, Pengfei Ren, Yating Zheng, Haofei Wang

**Affiliations:** 1 Department of Thoracic Surgery, Nanfang Hospital, Southern Medical University, Guangzhou, China; 2 Medical Department, 3D Medicines Inc., Shanghai, China

**Keywords:** immunotherapy, intratumoral heterogeneity, non-small cell lung cancer, postoperative recurrence, predictive

## Abstract

**Background:**

Intratumoral heterogeneity (ITH) is associated with poor prognosis in various solid tumors. However, its use as a predictive biomarker for immunotherapy in advanced non-small cell lung cancer (NSCLC) is still lacking.

**Methods:**

We used Maftools to analyze somatic variants of NSCLC from independent cohorts (POPLAR, OAK, Hellmann2018) including 504 patients with advanced NSCLC. Additionally, 121 patients (in-house cohort) with NSCLC who received radical treatment and underwent 733-panel NGS testing of surgical samples were included. We used Shannon entropy to measure ITH, analyzed correlation with immunogenic markers, and explored ITH’s prognostic effect on immunotherapy and its role in predicting postoperative recurrence risk. Using the TCGA cohort, we compared genetic characteristics between low ITH (ITH-L) and high ITH (ITH-H) groups, assessed mutational profiles, and evaluated associations between ITH scores and immune cell populations.

**Results:**

In the Hellmann2018 cohort, ITH-L correlated with higher durable clinical benefit (DCB) rate, objective response rate (ORR), and median progression-free survival (mPFS), highlighting ITH-L as a potential positive predictor for ICI therapy. Combining ITH with TMB revealed that ITH-L/TMB-H patients had significantly better DCB, ORR, and mPFS, supporting the potential predictive value of ITH when evaluated together with TMB. Validation in POPLAR/OAK cohorts supported the association of ITH, alone or combined with TMB, with immunotherapy outcomes. ITH-L patients exhibited higher DCB, ORR, longer mPFS, and median overall survival (mOS). Combining ITH and circulating tumor DNA validated these findings. In early-stage surgical NSCLC patients, ITH-L was associated with significantly higher median recurrence-free survival (mRFS). Mechanistically, the ITH-L group showed elevated TMB, neoantigen (SNV), and subclonal genome fraction, alongside reduced HRD scores and CNV burden. Mutational analysis identified XIRP2 as the most frequently altered gene, enriched in the ITH-L group, while SHOX2 was the only gene with higher frequency in the ITH-H group. Immune infiltration analysis revealed significant differences in M1 macrophages, activated memory CD4+ T cells, and naive CD4+ T cells between groups.

**Conclusions:**

ITH may serve as a potential biomarker associated with immunotherapy outcomes in advanced NSCLC and postoperative recurrence risk in early-stage surgical patients.

## 1 Introduction

Lung cancer, particularly non-small cell lung cancer (NSCLC), remains a leading cause of cancer-related mortality globally, despite advancements in diagnostic and therapeutic strategies. NSCLC accounts for approximately 85% of all lung cancer cases, with a majority being diagnosed at an advanced stage, underscoring the urgent need for effective treatment modalities (1). In recent years, the advent of immunotherapy, especially immune checkpoint inhibitors (ICIs) targeting the PD-1/PD-L1 axis, has revolutionized the treatment landscape of advanced NSCLC. However, the clinical response to ICIs is highly variable, prompting a deeper investigation into predictive biomarkers and underlying mechanisms driving this variability (2).

Among the factors influencing treatment efficacy, tumor mutational burden (TMB) and ITH have garnered significant attention. TMB, defined as the total number of somatic mutations per megabase of the tumor genome, has emerged as a potential biomarker for predicting response to ICIs. High TMB is associated with increased neoantigen load, enhancing the immunogenicity of tumors and potentially leading to better immunotherapy outcomes (3). However, the predictive power of TMB alone is limited, necessitating the incorporation of additional factors such as ITH.

ITH, characterized by the presence of distinct subclonal populations within the same tumor, adds another layer of complexity to cancer progression and treatment response (4). Studies using multiregion sequencing have revealed extensive ITH in NSCLC, which can drive differential responses to therapies and contribute to treatment resistance (5, 6). Specifically, in the context of immunotherapy, ITH-H may lead to heterogeneous expression of neoantigens and immune-evasion mechanisms, potentially diminishing the efficacy of ICIs (7, 8).

Furthermore, the role of ITH in early-stage NSCLC is equally critical. Surgical resection remains the primary curative intervention for early-stage NSCLC; however, postoperative recurrence rates are significantly high. Understanding the extent and nature of ITH pre- and post-surgery may help inform postoperative risk stratification, with evidence suggesting that high ITH levels are associated with poor recurrence-free survival and overall prognosis (9–11). By stratifying patients based on ITH, clinicians can tailor adjuvant therapies more effectively to tackle residual subclonal populations that may drive relapse.

This study aims to elucidate the combined predictive value of TMB and ITH on the efficacy of immunotherapy in advanced NSCLC and the impact of ITH on recurrence risk in early-stage patients. By leveraging comprehensive genomic profiling and clinical data, we seek to provide insights that could provide insights for future biomarker development for NSCLC patients.

## 2 Methods

### 2.1 Patients

We retrospectively collected a total of 504 NSCLC patients from three independent cohorts consisting of POPLAR, OAK and Hellmann2018 cohort, that were retrieved from published articles.

1. The POPLAR/OAK cohort included 429 patients with previously treated advanced NSCLC from the phase II POPLAR trial (NCT01903993) and the phase III OAK trial (NCT02008227), both of which evaluated atezolizumab versus docetaxel. All patients in the POPLAR/OAK trial implemented a genomic profiling of ctDNA with Foundation One panel (315-gene panel, 1.1Mb). bTMB values were acquired by NGS algorithm both in OAK and POPLAR cohort, PD-L1 expression on tumor cells or immune cells were evaluated by VENTANA PD-L1(SP142) assay only in OAK cohort. For analyses of immunotherapy efficacy, patients receiving atezolizumab with available response or survival data were evaluated.
2. The Hellmann2018 cohort consisted of 75 NSCLC patients who were treated with immune checkpoint inhibitors, and tumor somatic mutation data were obtained from whole-exome sequencing (12).

The in-house cohort included 121 patients with histologically confirmed NSCLC who underwent R0 radical resection and 733-gene panel sequencing of surgical tumor samples at Nanfang Hospital of Southern Medical University. Inclusion criteria were as follows: age 18–80 years, histologically confirmed NSCLC, R0 radical resection, available surgical tumor tissue for NGS testing, and no neoadjuvant therapy before surgery. Exclusion criteria included age <18 or >80 years, neoadjuvant therapy before surgery, and ECOG performance status >2. Recurrence-free survival (RFS) was defined as the time from R0 resection to disease recurrence or death from any cause. Recurrence was assessed based on postoperative clinical follow-up and imaging evaluation, with pathological confirmation when available.

The study was conducted following the declaration of Helsinki and was approved by the Ethical Review Board of Nanfang Hospital of Southern Medical University.

### 2.2 DNA sequencing, data processing, and variant calling (for tissue-based testing)

The captured libraries were loaded onto a NovaSeq 6000 platform (Illumina) for 100 bp paired-end sequencing with a mean sequencing depth of 35000X using a panel covering the exons of 733 cancer-related genes (**Supplementary Table 1**).

Raw data of paired samples (an FFPE sample and its normal tissue control) were mapped to the reference human genome hg19 using the Burrows-Wheeler Aligner (v0.7.12). PCR duplicate reads were removed and sequence metrics were collected using Picard (v1.130) and SAMtools (v1.1.19), respectively. Variant calling was performed only in the targeted regions. Somatic single nucleotide variants (SNVs) were detected using an in-house developed R package to execute a variant detection model based on binomial test. Local realignment was performed to detect indels. Variants were then filtered by their unique supporting read depth, strand bias, base quality as previously described. All variants were then filtered using an automated false positive filtering pipeline to ensure sensitivity and specificity at an allele frequency (AF) of ≥ 1%. Single-nucleotide polymorphism (SNPs) and indels were annotated by ANNOVAR against the following databases: dbSNP (v138), 1000Genome and ESP6500 (population frequency > 0.015). Only missense, stopgain, frameshift and non-frameshift indel mutations were kept. These retained somatic SNVs/indels were used for downstream ITH calculation. Copy number variations (CNVs) and gene rearrangements were detected as described previously.

### 2.3 Calculation of ITH using shannon entropy

ITH was quantified using the Shannon entropy based on the allele-frequency distribution of retained somatic SNVs/indels. For the in-house cohort, retained somatic SNVs/indels from the 733-gene panel sequencing pipeline described above were used. For public cohorts, processed somatic mutation matrices and clinical annotations were obtained from the original studies or public resources and used according to the original cohort-specific mutation-calling and filtering criteria. Because raw sequencing files or BAM-level data were not uniformly available across all public cohorts, these cohorts were not reprocessed from raw sequencing files.

For each sample, mutation allele frequencies were divided into 10 bins. Shannon entropy was calculated as H =-Σ P(f) log2[P(f)], where P(f) represents the proportion of mutations located in the f-th allele-frequency bin (13). Higher Shannon entropy indicates a broader and more even distribution of mutation allele frequencies, reflecting higher intratumoral genetic heterogeneity. This calculation was adapted from previous studies using Shannon entropy or Shannon Diversity Index to quantify genomic heterogeneity from MAF/VAF distributions.

All computational analyses were performed in R (version 4.0.2) and Python (version 3.8). Statistical analyses were also conducted to confirm the robustness of the Shannon entropy in reflecting ITH.

### 2.4 Correlation of ITH with PFS, OS, ORR, and DCB in the POPLAR, OAK, and Hellmann2018 cohorts

ITH was calculated for 504 patients in total, based on their the allele-frequency distribution of retained somatic SNVs/indels. Patients were stratified into ITH-L and ITH-H groups using the cohort-specific median ITH value as the cutoff. ICI efficacy analyses were performed using patients treated with immune checkpoint inhibitor monotherapy and with available response or survival data.

### 2.5 Correlation analysis between ITH and prognosis and adjuvant therapy in NSCLC patients undergoing radical surgery in in-house cohort

ITH was calculated for 121 patients in total, based on the allele-frequency distribution of retained somatic SNVs/indels. Patients were stratified into ITH-L and ITH-H groups using the cohort-specific median ITH value as the cutoff.

### 2.6 Correlative analysis of ITH with genomic features and immune cell infiltration

Based on the TCGA cohort, genetic characteristics between the ITH-L and ITH-H groups was compared, including TMB, homologous recombination deficiency (HRD), CNV burden, tumor purity, neoantigen, and subclonal genome fraction. The spectrum of genetic alterations and the mutational profile of significantly differentially expressed genes (p ≥ 0.05) was assessed between two groups. Additionally, RNA-Seq data from the TCGA cohort was employed to explore the correlation between ITH scores and immune cell infiltration levels (14).

### 2.7 Statistical analysis

Categorical variables were evaluated with Fisher’s exact tests. Unpaired Mann–Whitney U test was used to compare differences for continuous variables between groups. Correlation analysis was assessed by Pearson coefficient. ROC analysis was done using the ROCR package in R. Significance of overall survival (OS) and PFS was determined via Kaplan-Meier analysis with log-rank analysis. The hazard ratio was calculated by the coxph function of the survival package in R. Mann-Whitney U test and Chi-square test was employed to examine the statistical difference between the two groups. The scores of the 22 leukocyte subsets was estimated using the CIBERSORT algorithm. All statistical analysis was performed in the R statistical environment version 3.6.1. All tests were two-tailed and p-value < 0.05 was considered significant.

## 3 Results

### 3.1 ITH alone can predict the efficacy of immunotherapy in Hellmann2018 cohort

**Figure 1:**
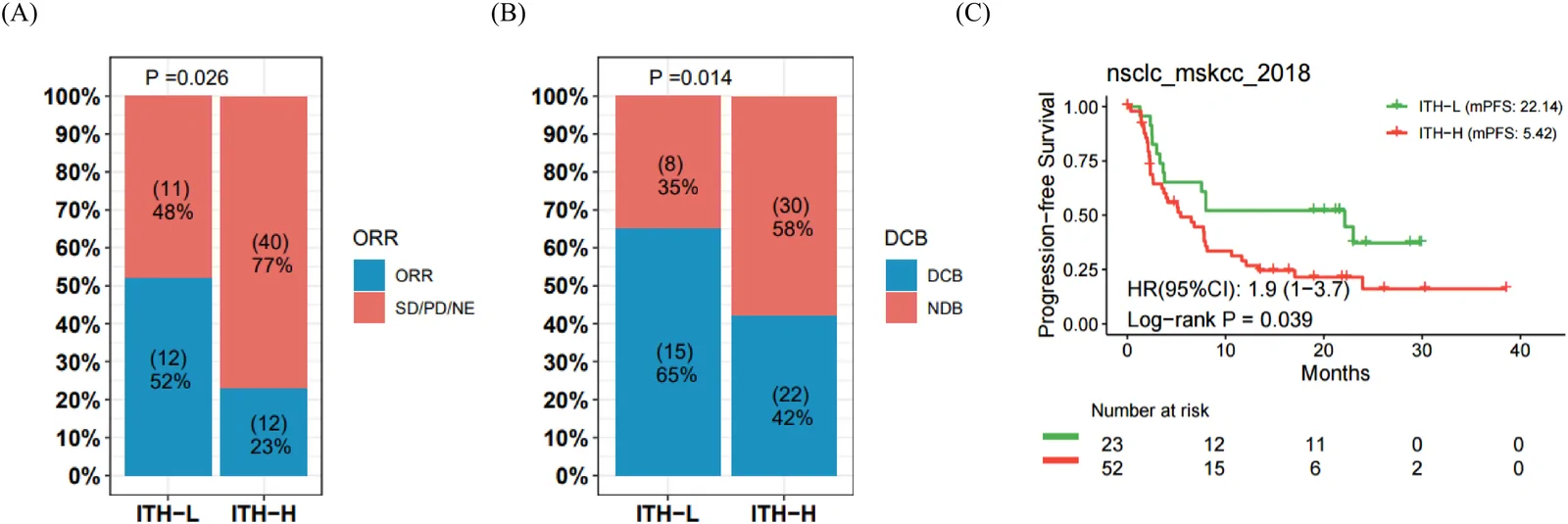
Predictive value of ITH for immunotherapy efficacy in the Hellmann 2018 cohort. **(A)** Impact of ITH level on ORR; **(B)** Impact of ITH level on DCB; **(C)** Impact of ITH level on PFS.

The ITH level for each patient was calculated and shown **Supplementary Table 2**. A higher ORR rate, DCB rate and mPFS were significant correlated with patients with ITH-L than ITH-H (ORR rate, 52% vs 23%, P = 0.026; DCB rate, 65% vs 42%, P = 0.014; mPFS, 22.14 vs 5.42 months, log-rank p = 0.039, HR = 1.9 (95% CI, 1–3.7); **Figures 1A–C**). These findings indicated that low level of ITH might be a positive predictor for ICI therapy.

### 3.2 ITH combined with TMB can predict the efficacy of immunotherapy in Hellmann2018 cohort

**Figure 2:**
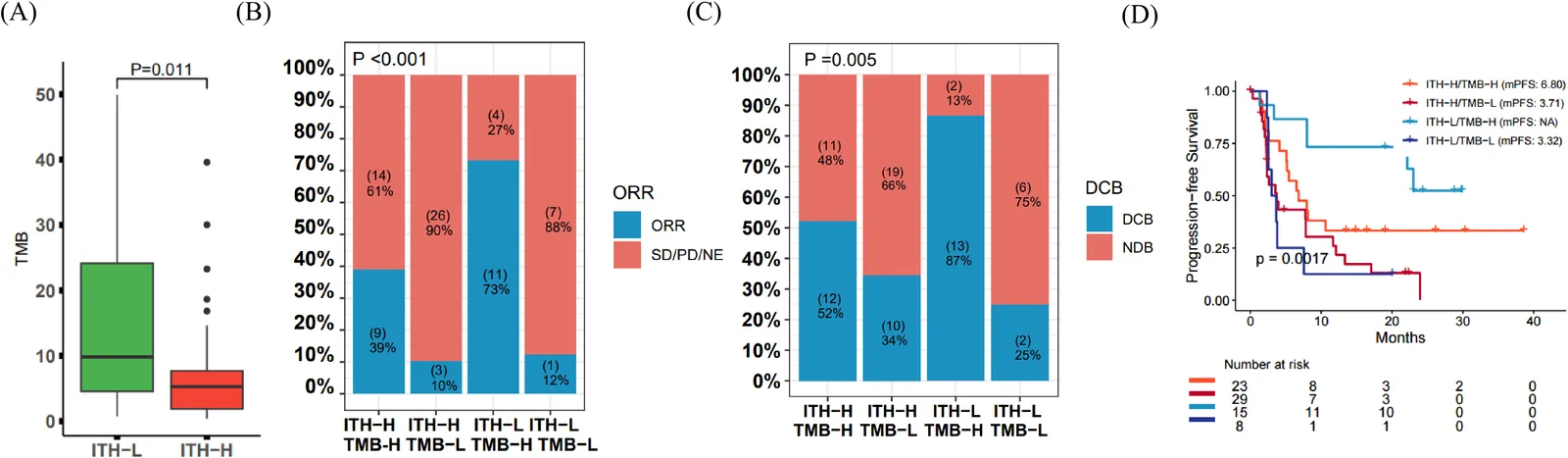
Predictive value of ITH combined with TMB (TMB grouped by median value) for immunotherapy efficacy in the Hellmann 2018 cohort. **(A)** TMB distribution in different ITH levels; **(B)** Impact of different subgroups on ORR; **(C)** Impact of different subgroups on DCB; **(D)** Impact of different subgroups on PFS.

There was significant difference in TMB levels between ITH-L and ITH-H groups (P = 0.011, **Figure 2A**). When the high and low levels of TMB are divided according to the median value, most of TMB-H patients were ITH-L (**Supplementary Table 2**). In the TMB-H group, a significantly higher DCB rate, ORR rate and mPFS were observed in patients with ITH-L (ORR rate, 73% vs 39%, P < 0.001; DCB rate, 87% vs 52%, P = 0.005; mPFS, not reached vs 6.8 months, P = 0.0017; **Figures 2B–D**). In addition, No such significant difference was observed in the TMB-L group. A pooled analysis of ITH and TMB show that DCB rate, ORR rate and mPFS in the ITH-L+TMB-H group was significantly better than that in the ITH-H+TMB-H, ITH-L+TMB-L, ITH-H+TMB-L group (ORR rate, P < 0.001; DCB rate, P = 0.005; mPFS, P = 0.0017; **Figures 2B–D**).

All of the above results held true when the TMB levels were grouped by 10mut/MB (**Supplementary Figure 1**).These findings supported the potential predictive value of ITH when interpreted together with TMB. These findings indicated that patients more likely to benefit from immunotherapy may be better identified by using the combination of ITH and TMB.

### 3.3 Validation in the POPLAR/OAK cohorts

**Figure 3:**
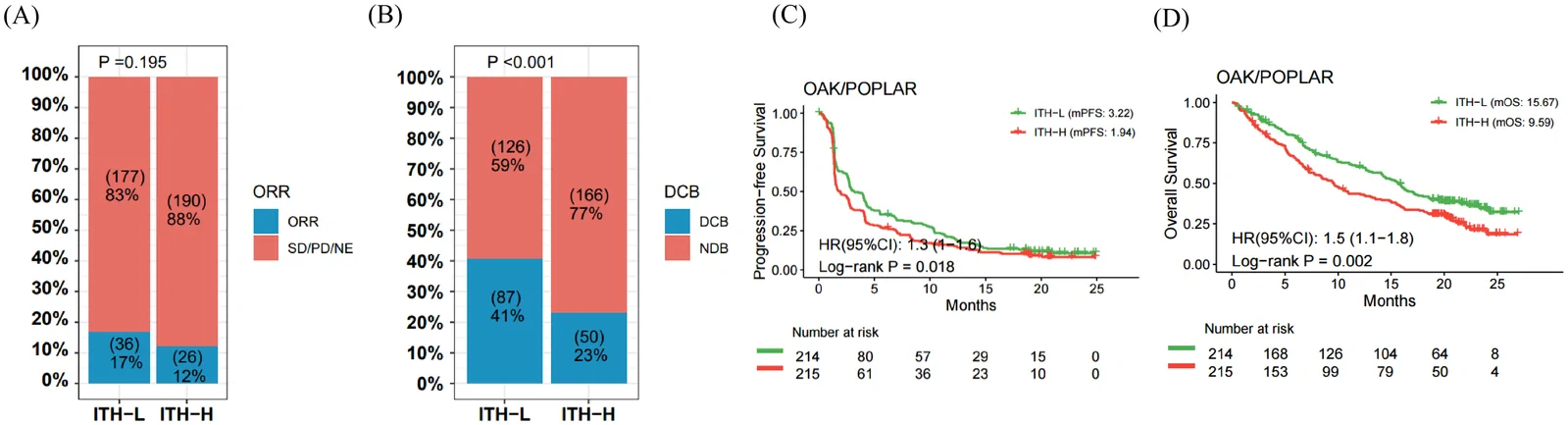
Predictive value of ITH for immunotherapy efficacy in the POPLAR/OAK cohort. **(A)** Impact of ITH level on ORR; **(B)** Impact of ITH level on DCB; **(C)** Impact of ITH level on PFS; **(D)** Impact of ITH level on OS.

**Figure 4:**
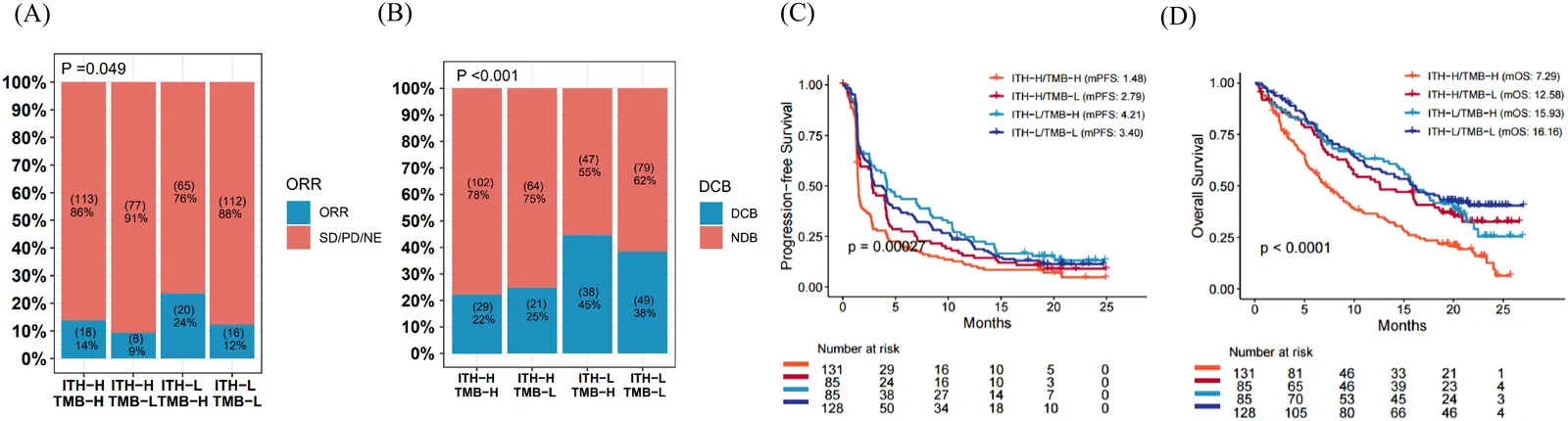
Predictive value of ITH combined with TMB (TMB grouped by median value) for immunotherapy efficacy in the POPLAR/OAK cohort. **(A)** Impact of different subgroups on ORR; **(B)** Impact of different subgroups on DCB; **(C)** Impact of different subgroups on PFS; **(D)** Impact of different subgroups on OS.

Our results were also validated in external validation cohorts. We found that ITH could still effectively predict the efficacy of immunotherapy in POPLAR/OAK cohorts alone or combined with TMB (**Figures 3**, **4**).

Precious tumor biopsy specimens may be exhausted from routine clinical tests. In order to investigate the prediction role of ITH in addition to the WES platform, we applied the ITH measurement algorithm to circulating tumor DNA (POPLAR, OAK) data. In POPLAR/OAK cohorts, patients with ITH-L showed significantly higher ORR rate, DCB rate and longer mPFS, mOS in POPLAR/OAK (ORR rate, 17% vs 12%, P = 0.195; DCB rate, 42% vs 23%, P < 0.001; mPFS, 3.22 vs 1.94 months, log-rank p = 0.018, HR = 1.3 (95% CI, 1–1.6); mOS, 15.67 vs 9.59 months, log-rank p = 0.002, HR = 1.5 (95% CI, 1–1.8); **Figures 3A–D**). Further analysis found that patients in when the high and low levels of TMB are divided according to the median value, most of bTMB-H patients were ITH-L (**Supplementary Table 2**). In the TMB-H group, a significantly higher ORR rate, DCB rate, mPFS and mOS were observed in patients with ITH-L (ORR rate, 24% vs 14%, P = 0.049; DCB rate, 45% vs 22%, P <0.001; mPFS, 4.21 vs 1.48 months, log-rank p = 0.00927; mOS, 15.93 vs 7.29 months, log-rank p < 0.0001; **Figures 4A–D**). In addition, a significantly higher ORR rate, DCB rate, mPFS and mOS were observed in patients with ITH-L in the TMB-L group (ORR rate, 12% vs 9%, P = 0.049; DCB rate, 38% vs 25%, P <0.001; mPFS, 3.40 vs 2.79 months, log-rank p = 0.00927; mOS, 16.16 vs 12.58 months, log-rank p < 0.0001; **Figures 4A–D**). A pooled analysis of ITH and TMB show that ORR rate, DCB rate, mPFS and mOS in the ITH-L+TMB-H group was significantly better than that in the ITH-H+TMB-H, ITH-L+TMB-L, ITH-H+TMB-L group (ORR rate, P = 0.049; DCB rate, P <0.001; mPFS, log-rank p = 0.00927; mOS, log-rank p < 0.0001; **Figures 4A–D**).

All of the above results held true when the TMB levels were grouped by 10mut/MB (**Supplementary Figure 2**).

This finding not only further validated the robustness of ITH in predicting clinical benefit of immunotherapy in NSCLC, but also benefit the future promotion and application of the ITH index with economical, versatile, and noninvasive methods.

### 3.4 Evaluation of the association between ITH and postoperative recurrence and prognosis

To explore the impact of ITH on recurrence and survival outcomes of NSCLC patients after radical surgery, 121 patients (in-house cohort) with NSCLC who received radical treatment and underwent 733-gene panel NGS testing of surgical samples at Nanfang Hospital of Southern Medical University were included in this study. The median follow-up time exceeded 60 months based on the currently available follow-up records. Baseline clinicopathologic characteristics and genomic profile are summarized in **Supplementary Table 3**.

Distinct differences in tumor prognostic indicators were observed between the ITH-L and ITH-H groups. The analysis showed that a total of 20 patients experienced recurrence, with 5 in the ITH-L group and 15 in the ITH-H group. The difference in recurrence rates between the two groups was statistically significant (p = 0.016), indicating that ITH-H patients had a substantially higher risk of recurrence than ITH-L patients. Furthermore, a greater proportion of ITH-H patients received postoperative adjuvant therapy, suggesting a potentially worse prognosis for these individuals (**Table 1**). Further analysis focusing on Stage I patients revealed 3 recurrences in the ITH-L group and 9 in the ITH-H group. However, the difference in recurrence rates between two groups did not reach statistical significance (p = 0.070) (**Table 2**).

**Table 1:** Comparison of baseline characteristics of patients with different levels of ITH.

Factor	ITH-L (n=60)	ITH-H (n=61)	P value
Age	60.92 ± 10.72	63.97 ± 8.58	0.085
Sex			0.526
Female	31 (51.67%)	28 (45.90%)	
Male	29 (48.33%)	33 (54.10%)	
Survival			0.174
Yes	58 (96.67%)	54 (88.52%)	
No	2 (3.33%)	7 (11.48%)	
Recurrence			0.016
No	55 (91.67%)	46 (75.41%)	
Yes	5 (8.33%)	15 (24.59%)	
TNM stage			0.388
I	51 (85.00%)	48 (78.69%)	
II	5 (8.33%)	10 (16.39%)	
III	4 (6.67%)	3 (4.92%)	
T stage			0.745
1	49 (81.67%)	46 (75.41%)	
2	10 (16.67%)	14 (22.95%)	
3	1 (1.67%)	1 (1.64%)	
N stage			0.804
0	27 (45.00%)	31 (50.82%)	
1	27 (45.00%)	25 (40.98%)	
2	6 (10.00%)	5 (8.20%)	
Adjuvant therapy			0.035
Yes	22 (36.67%)	34 (55.74%)	
No	38 (63.33%)	27 (44.26%)	

**Table 2:** Analysis of recurrence in stage I patients with different ITH levels.

Factor	ITH-L (n=49)	ITH-H (n=50)	P value
Recurrence			0.070
No	46 (93.88%)	41 (82.00%)	
Yes	3 (6.12%)	9 (18.00%)	

**Figure 5:**
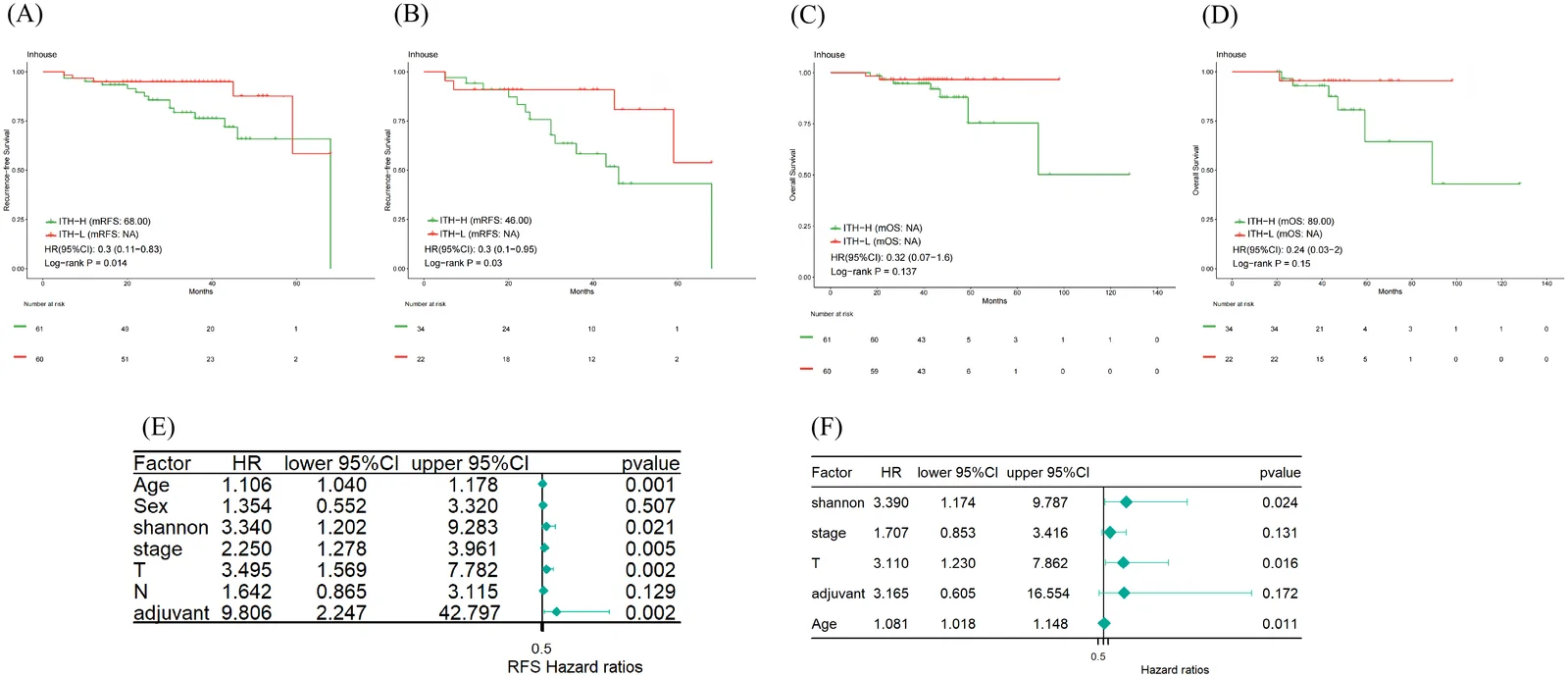
Impact of different ITH levels on postoperative survival outcomes in NSCLC patients after R0 resection in the internal cohort. **(A)** RFS by ITH level in the overall population; **(B)** RFS by ITH level in patients receiving adjuvant therapy; **(C)** OS by ITH level in the overall population; **(D)** OS by ITH level in patients receiving adjuvant therapy; **(E)** Univariate Cox regression analysis of postoperative recurrence; **(F)** Multivariate Cox regression analysis of postoperative recurrence.

In the general population, we found that a significantly higher mRFS was correlated with patients with ITH-L [mRFS, not reached vs 68 months, log-rank p = 0.014, HR = 0.3 (95% CI, 0.11-0.83); **Figure 5A**]. However, the postoperative mOS did not differ significantly between the ITH-L and ITH-H groups [not reached vs not reached, log-rank p = 0.137, HR = 0.32 (95% CI, 0.07–1.6); **Figure 5C**]. Nevertheless, the Kaplan-Meier curve indicated a favorable survival trend in the ITH-L group compared to the ITH-H group.

Among patients who received adjuvant therapy, we also assessed the recurrence risk and survival benefit between those with ITH-L and ITH-H. It was found that patients with ITH-L still exhibited significantly longer mRFS compared to those with ITH-H (not reached vs 46 months, log-rank p = 0.03; HR = 0.3 (95% CI, 0.1–0.95); **Figure 5B**). Regarding clinical outcomes, the mOS was not reached in the ITH-L group, while it was 89 months in the ITH-H group, with no significant difference between two groups [log-rank p = 0.15, HR = 0.24 (95% CI, 0.03–2); **Figure 5D**]. Of note, the survival trend in the adjuvant-treated cohort was consistent with that of the general population, favoring patients with ITH-L.

Then univariable Cox regression analysis for postoperative recurrence was conducted, revealing age, ITH, stage, T stage, and receipt of adjuvant therapy as significant risk factors for recurrence in NSCLC. Notably, ITH-H was associated with a significantly increased risk of tumor recurrence [HR = 3.340 (95% CI, 1.202–9.283), p = 0.021; **Figure 5E**]. Subsequent multivariable Cox analysis incorporating the above significant factors was performed. Patients with ITH-H maintained an increased risk of recurrence compared to those with ITH-L [HR = 3.390 (95% CI, 1.174–9.787), p = 0.024], while older age and advanced T stage were also significant independent risk factors (**Figure 5F**).

### 3.5 Association of ITH with genetic profiles and immune cell infiltration

**Figure 6:**
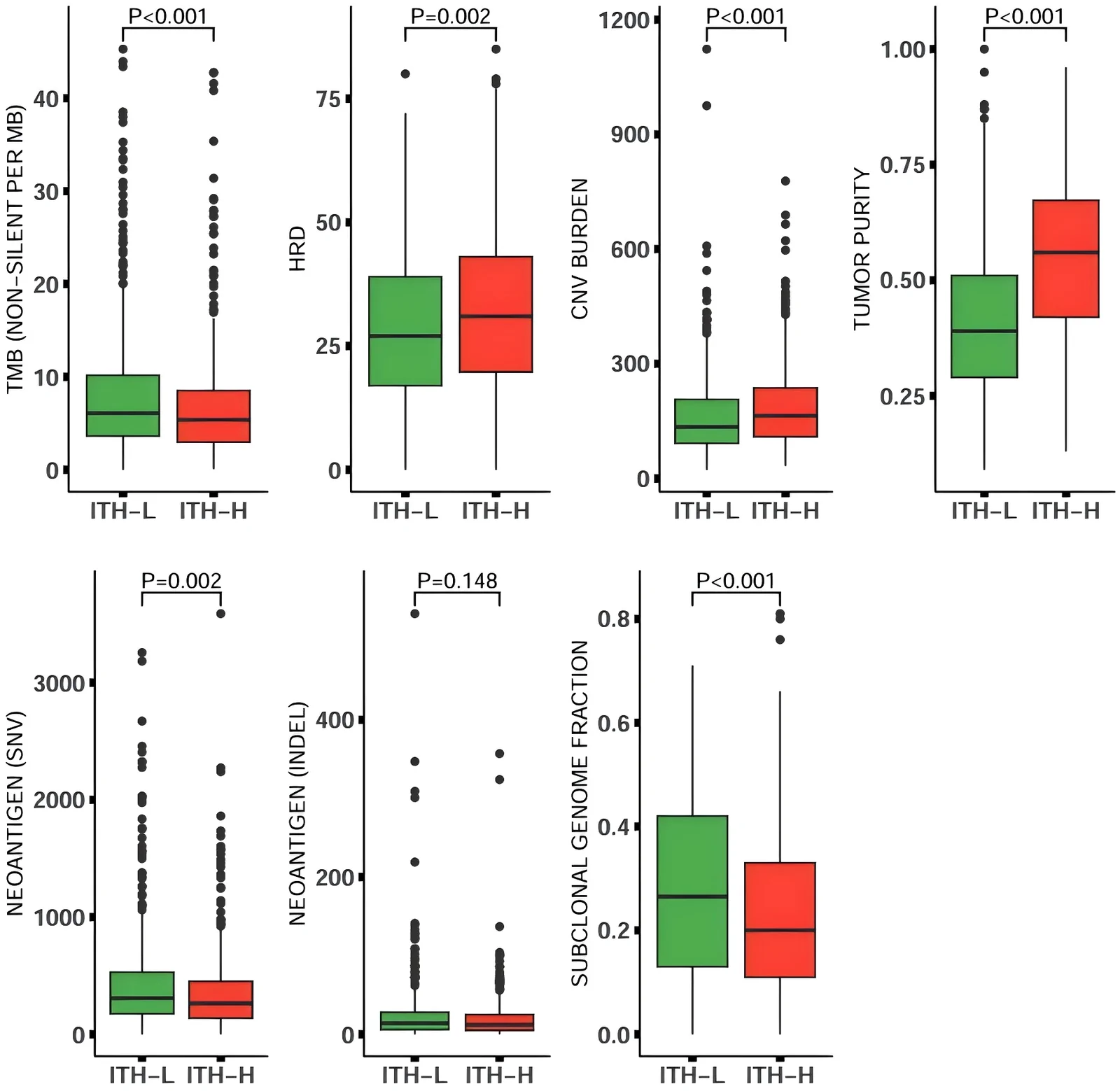
Correlation analysis between ITH level and genomic features.

Regarding genetic characteristics, the ITH-L group exhibited significantly higher TMB (P < 0.001), neoantigen (SNV, P = 0.002), and subclonal genome fraction (P < 0.001). Conversely, patients with ITH-L tended to have lower HRD scores (P = 0.002), CNV burden (P < 0.001), and tumor purity (P < 0.001) (**Figure 6**). These reported genomic feature differences remained significant after conservative Benjamini-Hochberg FDR adjustment based on the nominal P values.

**Figure 7:**
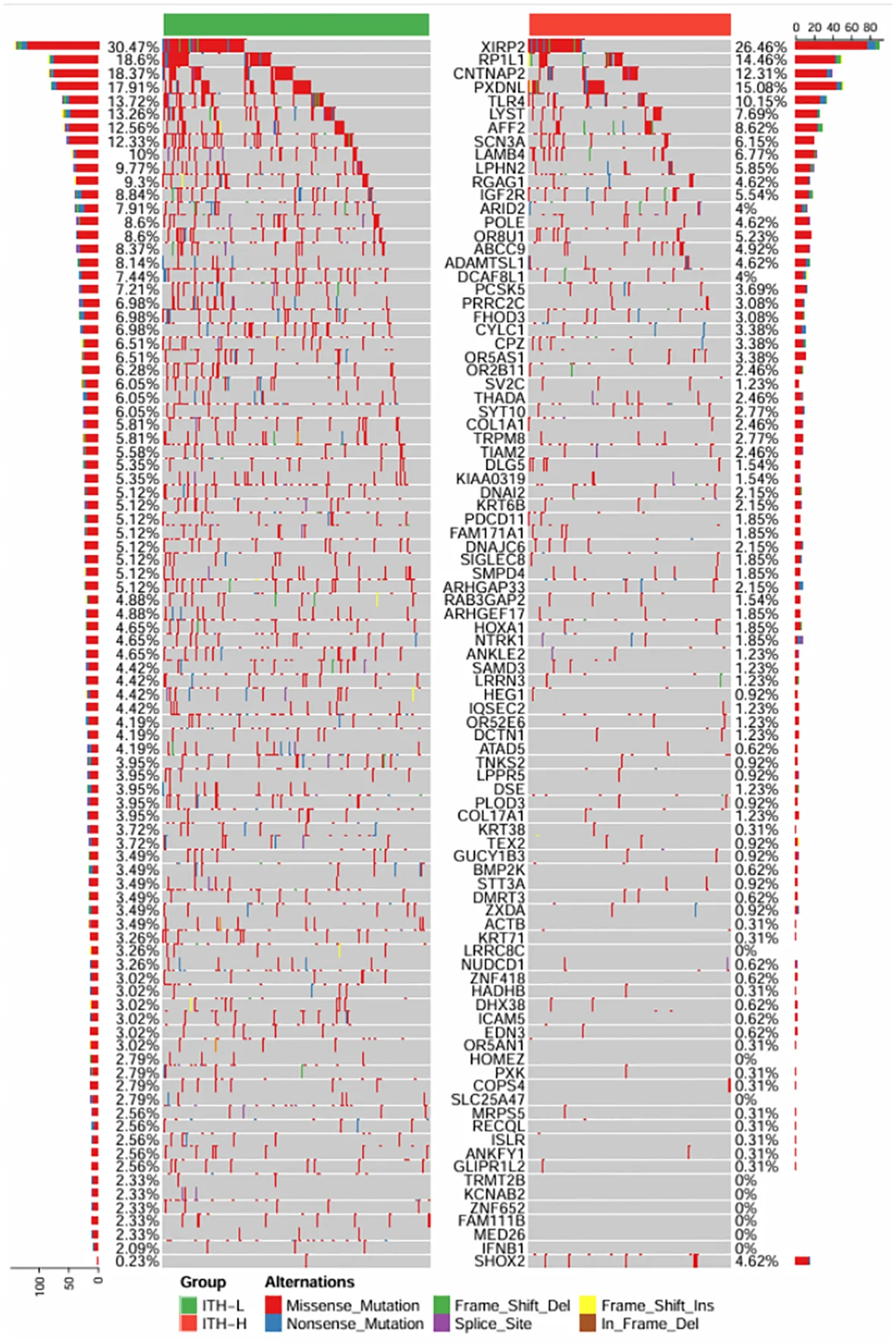
Mutation landscape of significantly differentially mutated genes in different ITH levels.

The genetic alteration profiles were further analyzed between two groups. It was revealed that missense mutation was the most common type of alteration. Among the significantly differentially expressed genes, nearly all genes exhibited a higher mutation frequency in the ITH-L group. The gene with the highest frequency was XIRP2, altered in 30.47% in the ITH-L group compared to 26.46% in the ITH-H group. SHOX2 was the only gene with a higher frequency in the ITH-H group (4.62% vs. 0.23%) (**Figure 7**).

**Figure 8:**
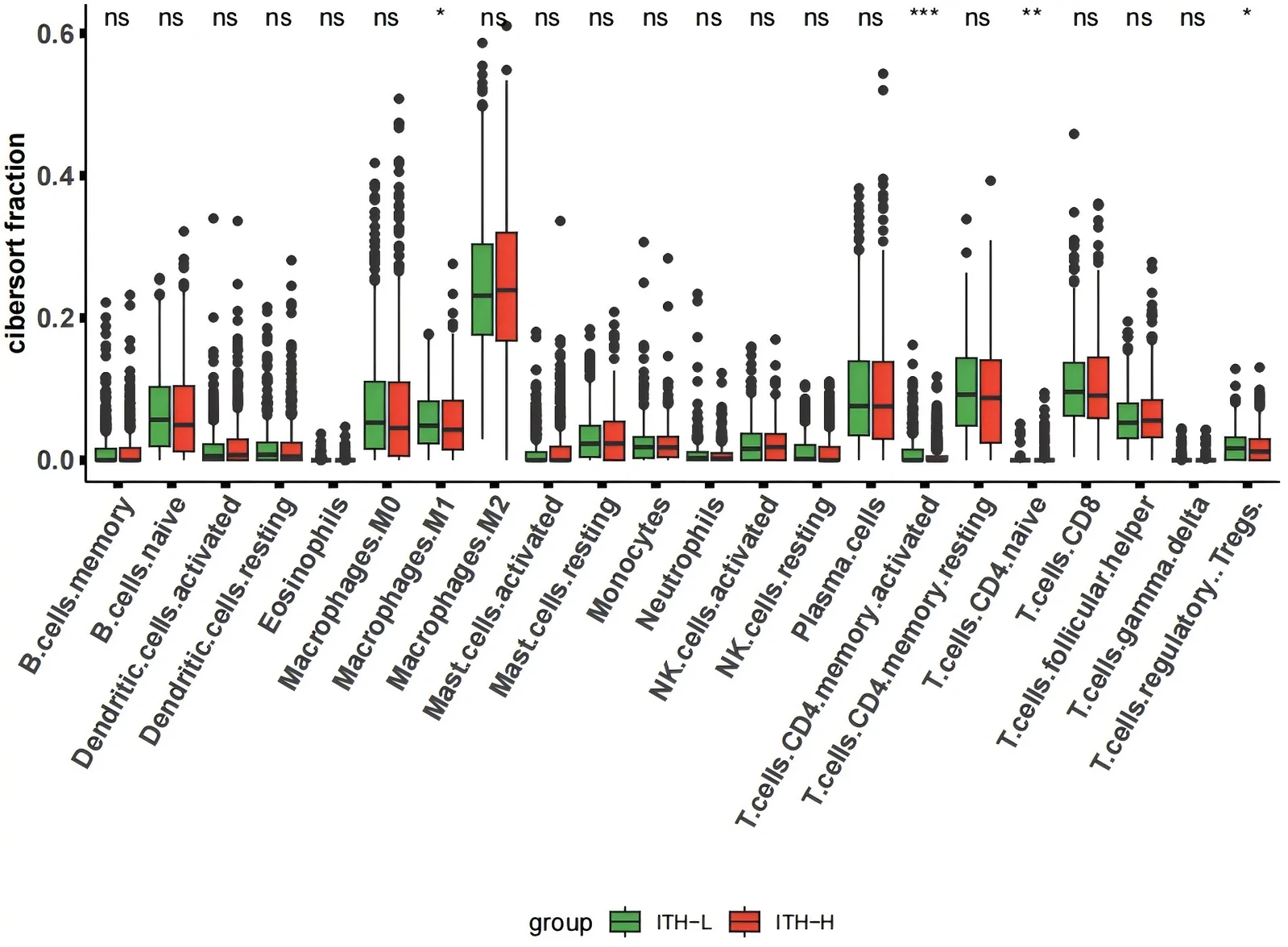
Analysis of immune cell infiltration levels in different ITH groups. ns, not statistically significant; *: p<0.05; **: p<0.01; ***: p<0.001.

Immune infiltration analysis was performed to investigate the relationship between ITH and prognosis. Significant differences were observed between two groups in the proportions of M1 macrophages, activated memory CD4+ T cells, naive CD4+ T cells, and regulatory T cells (Tregs), with the ITH-L group showing greater infiltration. These findings suggested that patients with ITH-L may derive greater benefit from ICIs therapy (**Figure 8**).

## 4 Discussion

This study underscores the critical role of ITH in predicting the efficacy of ICIs in NSCLC. Our data demonstrates that ITH was associated with immunotherapy outcomes, particularly when evaluated together with TMB.

In analyzing the Hellmann2018 cohort, we observed that patients with ITH-L exhibited higher DCB, ORR, and mPFS. These results align with the notion that lower genetic diversity within tumors may be associated with better immune recognition and response, potentially due to more uniform expression of neoantigens (15, 16). The integration of ITH with TMB further refined our ability to predict clinical outcomes. Notably, TMB-H patients predominantly exhibited lower ITH levels (ITH-L), and within this TMB-H group, ITH-L correlated with significantly better clinical outcomes. This suggests that ITH and TMB may provide complementary, although partially overlapping, predictive information. Indeed, pooled analysis revealed that the ITH-L and TMB-H subgroup had superior DCB, ORR, and mPFS compared to other combinations, supporting the potential value of this dual-biomarker framework for immunotherapy stratification (17). These findings are consistent with existing literature that highlights the importance of tumor heterogeneity and mutational burden in cancer treatment outcomes. Studies have shown that high TMB is predictive of better response to PD-1/PD-L1 inhibitors, likely due to the higher load of neoantigens presented to the immune system (18, 19). Similarly, lower ITH is believed to reduce immune evasion mechanisms, thereby improving response rates and survival outcomes (20).

External validation in the POPLAR and OAK cohorts confirmed the robustness of ITH as a predictive marker when assessed via circulating tumor DNA (ctDNA). Patients with ITH-L had improved DCB, ORR, mPFS, and mOS. These results emphasize the feasibility of using non-invasive methods to determine ITH, which is advantageous for clinical implementation given the limited availability of biopsy tissue (21, 22). However, ctDNA-derived ITH may be influenced by tumor shedding dynamics, tumor burden, metastatic site contribution, assay sensitivity, and sequencing depth; therefore, it should not be interpreted as a direct equivalent of spatial intratumoral architecture in tissue (23).

Furthermore, ITH was also associated with recurrence risk in operable early-stage NSCLC. In our investigation, when early-stage patients were stratified based on ITH, a statistically difference was identified in the number of recurrence events. Although no significant difference in recurrence was detected between two groups within the Stage I population, a higher recurrence rate was observed in the ITH-H group. This finding suggested that ITH assessment may help identify Stage I patients at potentially higher recurrence risk, although its role in guiding adjuvant therapy requires further validation. Nonetheless, due to the limited sample size of this cohort, this conclusion warranted further validation in larger prospective studies.

Additionally, this study revealed that the ITH-L patients had a higher mRFS, and this trend remained consistent among those who received postoperative adjuvant therapy. Elevated ITH was reported to be correlated with an increased risk of recurrence in small cell lung cancer (SCLC), hepatocellular carcinoma, breast cancer, supporting its potential role as a biomarker for predicting tumor recurrence risk (24–26). Although no statistically difference was observed in mOS between two groups, the ITH-L group demonstrated a more favorable survival curve trend. Since OS was generally prolonged after R0 resection, further extended follow-up was needed to determine whether ITH ultimately affected OS, and whether the inferior RFS in the ITH-H group translated into a difference in OS. The TRACERx study, which enrolled an early-stage operable NSCLC population, reported a marked association between elevated ITH in somatic copy number alterations and shorter disease-free survival (DFS) (27). Stanislav et al. also demonstrated that ITH-H predicted inferior survival outcomes (17). Stratification by ITH may help identify patients at higher recurrence risk, but its utility for tailoring adjuvant therapy strategies requires validation in larger prospective cohorts.

We explored genomic and immune-related features associated with ITH, which may reflect contributions from both genetic and non-genetic factors (28). Analysis of genetic characteristics revealed that the ITH-L group exhibited significantly higher TMB, neoantigen(SNV)and subclonal genome fraction, alongside lower HRD scores, CNV burden, and tumor purity. These biomarkers reflect different dimensions of tumor genomic architecture. TMB and neoantigen burden quantify the total number of somatic alterations and predicted immunogenic peptides, whereas Shannon entropy-based ITH reflects the breadth and evenness of mutation allele-frequency distributions. Thus, tumors with high TMB and neoantigen burden may still show low entropy-based ITH if a substantial proportion of mutations are concentrated in dominant clonal or near-clonal populations (9). Such a pattern may indicate a more uniformly immunogenic tumor architecture, which could facilitate immune recognition and response to immune checkpoint blockade (29). This may partly explain why ITH-L tumors showed better immunotherapy-related outcomes in our analysis. Previous studies reported that somatic mutations, CNV, and chromosomal instability drove genomic instability, thereby promoting cellular diversity and genetic ITH (30). Furthermore, our findings supported integrating ITH with TMB as a composite predictor for ICI response, rather than relying on ITH as a standalone metric. Although TMB has been widely investigated as an immunotherapy biomarker, its predictive performance varies across cancer types, assays, and clinical contexts, highlighting the need for complementary biomarkers (19, 31). Prior evidence indicated that ITH, either alone or in combination with TMB, could predict response to immunotherapy, despite the absence of a significant correlation between the two metrics (9). This combined approach may help improve the identification of patients likely to benefit from ICIs. Assessment of the mutational landscape identified XIRP2 as the most frequently mutated gene, with a significantly higher mutation frequency in the ITH-L group. Previous reports indicated that XIRP2 mutation was associated with increased TMB and neoantigen load in NSCLC, and patients carrying XIRP2 mutations demonstrated significantly improved PFS following ICI treatment (32). Therefore, the observed enrichment of XIRP2 mutations in the ITH-L group should be interpreted as an exploratory finding, and its biological and predictive relevance requires further validation.

The interplay between ITH and the TME played a critical role in driving tumor progression and therapeutic response (33). Our immune infiltration analysis was consistent with this possibility, showing significantly increased abundance of several immune cell populations in the ITH-L group. This observation was consistent with previous findings. Wang et al. reported a strong association between nine immune cell types and ITH status, and the infiltration scores of these immune cells decreased as ITH increased (34). These results indicated that ITH was associated with immune cell infiltration within the TME, which may be related to patient prognosis.

Future studies with extended follow-up and larger cohorts are essential to validate these findings and refine the integration of ITH and TMB into clinical workflows. In addition, because the immune infiltration analysis was based on computationally inferred CIBERSORT cell fractions and involved multiple immune cell subsets, these findings should be interpreted as exploratory evidence. Developing standardized, non-invasive assays for ITH assessment will be crucial for translating these insights into routine clinical practice.

## 5 Conclusion

In conclusion, our study highlights the significance of ITH as a potential predictor, particularly in combination with TMB, for immunotherapy efficacy in NSCLC. We elucidated the molecular mechanisms by which ITH influences the biological characteristics and tumor immune microenvironment. The findings support further evaluation of ITH, particularly in combination with TMB, as a potential biomarker for immunotherapy stratification in NSCLC. Moreover, the application of ITH assessment via ctDNA may provide a practical and non-invasive complement to tissue-based evaluation, but its clinical application requires further validation.

## Data Availability

The original contributions presented in the study are included in the article/**Supplementary Material**. Further inquiries can be directed to the corresponding author.
